# The tiger mosquito in Lebanon two decades after its introduction: A growing health concern

**DOI:** 10.1371/journal.pntd.0010206

**Published:** 2022-02-09

**Authors:** Nabil Haddad, Hayssam Omran, Fadila Amraoui, Renée Zakhia, Laurence Mousson, Anna-Bella Failloux

**Affiliations:** 1 Laboratory of Immunology and Vector-Borne Diseases, Faculty of Public Health, Lebanese University, Beirut, Lebanon; 2 Laboratory of Arboviruses and Insect Vectors, Department of Virology, Institut Pasteur, Paris, France; Imperial College London, UNITED KINGDOM

## Abstract

The tiger mosquito was introduced to the Eastern region of the Mediterranean basin more than twenty years ago. In Lebanon, it was first observed in 2002 in a limited number of locations mainly from the coastal area of the country. In the absence of national entomological control program, this invasive mosquito became an established species and is now considered in many localities, a source of nuisance because of its human biting behavior. Several entomological surveys were conducted to monitor the geographic spread and the seasonal dynamics of *Aedes albopictus* by collecting adult stages and by monitoring oviposition activity. Moreover, its susceptibility to the common groups of insecticides was assessed using WHO standard bioassays. Previous vector competence studies revealed that local strains were able to transmit Chikungunya and Dengue viruses. Due to the increased risk of Zika virus introduction in the country, we determined the competence of local populations to transmit this virus. Mapping results showed that *Ae*. *albopictus* is mainly spread in the relatively humid western versant of the Mount Lebanon chain reaching 1000m altitude, while it is absent from arid and semi-arid inland areas. Besides, this mosquito is active during 32 weeks from spring till the end of autumn. Local strains of the tiger mosquito are susceptible to pyrethroids and carbamates but resistant to organophosphates and organochlorines. They showed ability to transmit Zika virus; however, only 9% of females were capable to excrete the virus in their saliva at day 28 post infection. Current and previous observations highlight the need to establish a surveillance system in order to control this mosquito and monitor the potential introduction of related diseases.

## Author summary

*Aedes albopictus*, also called the tiger mosquito, is one the most invasive mosquito species worldwide. It is originated from South-East Asia and islands of the Pacific and Indian oceans and is known to be able to transmit to humans many viral diseases. During the last four decades it succeeded to invade many countries in all continents through an increasing international trade and travel. This mosquito was first observed in Lebanon, in the Middle East region, in 2002. Previous studies revealed that the introduced mosquito was able to transmit Chikungunya and Dengue viruses under experimental conditions. Therefore, it represents a health threat for the Lebanese population. In this study, the authors assessed the status of the tiger mosquito in the country two decades after its first record. They showed that it is widespread in the humid and sub-humid regions of Mount Lebanon chain and is active from spring through late autumn. The authors experimentally demonstrated the ability of this mosquito to transmit Zika virus, a virus that is highly likely to get introduced to Lebanon due to important population flow from South America to the country during the summer season. Finally, the authors found that local populations of tiger mosquito were susceptible to insecticides of the pyrethroids and carbamates groups but resistant to those of the organochlorines and organophosphates groups. The generated information should help national health authorities to establish a targeted surveillance and control strategies for this mosquito.

## Introduction

*Aedes albopictus* (Skuse, 1895) is originated from South-East Asia and islands of the Western Pacific and Indian Ocean, where it is considered a potential vector of several viral pathogens [1]. Since more than four decades, promoted by the increasing global trade and population movements, this species has invaded many countries around the world where it became adapted to urban, peri-urban and even, rural environments [2]. The tiger mosquito is considered one of the most invasive mosquito species worldwide [3]. In Europe, it has been reported from 27 countries and was established in 21 of them [4].

This mosquito is considered the natural vector of chikungunya virus (CHIKV) and in some regions, dengue virus (DENV) [1,2,5]. However, it has a much larger potential being experimentally able to transmit at least 26 arboviruses belonging to different families including *Flaviviridae*, *Togaviridae* and *Phenuiviridae* [6]. The tiger mosquito was responsible for first local cases of CHIKV and DENV in Europe: Italy, France and Croatia [7–11]. In the South of France, it was incriminated in the first autochthonous Zika virus (ZIKV) cases [12]. Control measures against this mosquito are mainly based on insecticide treatments. However, insecticide resistance is considered a serious challenge that jeopardizes the efficiency of vector control measures. *Ae*. *albopictus* has developed a marked resistance to four major insecticide classes mainly in Asia [13].

In the Middle East, the presence of *Ae*. *albopictus* is confirmed in countries on the Eastern side of the Mediterranean Sea, namely in Turkey, Syria, Lebanon, Palestine, Israel and Jordan [14–18]. Further East, this mosquito is present in Iran and Pakistan. Of all those countries, only in Pakistan and Turkey, the tiger mosquito is co-reported with *Aedes aegypti*, the primary vector of many arboviral diseases [19].The tiger mosquito was first observed in Lebanon in 2002 in a limited number of locations mainly from the coastal area of the country[15]. Since that time and in the absence of national entomological control program, this invasive mosquito has succeeded to expand its geographical distribution. In many localities, it became a source of nuisance because of its human biting behavior. Since the beginning of the civil war in 1975, national entomological control programs have ceased and did not resume despite the end of this war. Presently, control efforts are occasionally and randomly conducted by municipalities. They rely on the use of insecticides of different classes mainly pyrethroids. Meanwhile, their efficacy on the tiger mosquito and the potential existence of resistant populations has never been evaluated in the country.

The presence of *Ae*. *albopictus* in Lebanon is a potential risk for outbreaks of related arboviruses such as CHIKV, DENV and ZIKV. Of those, only DENV was reported from Lebanon. This virus used to be endemic before 1950 [20] until it disappeared following the implementation of the malaria eradication program which led to the elimination of *Aedes aegypti*, the prevailing vector at that time. In 2012, a patient from Aley (Mount Lebanon) with no prior travel history was diagnosed with dengue fever [21]. Moreover, a serological study conducted in 2013 among healthy patients revealed at least two sera (one belonging to a patient with no travel history) with high titers of neutralizing antibodies to DENV-2 suggestive of local circulation of DENV [22]. Local populations are competent to transmit DENV and CHIKV [23]. However, their capacity to transmit ZIKV is not yet assessed. The risk of introduction of these viruses into the country is highly likely considering the important number of Lebanese expatriates in South America, visiting the country during the summer.

Here we assess the geographic spread of this mosquito almost two decades after its first detection in the country and determine its seasonal dynamics. We also evaluated the susceptibility of Lebanese populations of *Ae*. *albopictus* to commonly used insecticides. Moreover, and due to the increased risk of ZIKV introduction, we determined the competence of local populations to transmit this virus.

## Methods

### Mapping of *Aedes albopictus*

In order to collect adult specimens of *Ae*. *albopictus*, BG-sentinel traps (associated with BG lure as attractants) were placed in several localities between May and October 2015 in order to cover different bioclimatic zones (arid, sub-humid and humid) and different districts over Lebanon in urban and rural environments. Each trap was left in the trapping site during at least 12 hours. In 2018, further collection campaigns were performed between June and September, using CDC traps baited with CO_2_ (dry ice source) in addition to BG-sentinel traps. Geographic coordinates of the trapping sites were recorded using GPS application of smart phones. Collected mosquitoes were brought to the laboratory for identification and reporting was made on the presence/absence of *Ae*. *albopictus* in each trapping location.

### Seasonal dynamics

In order to track the seasonal activity of *Ae*. *albopictus*, eggs were collected from two locations: Fanar (Eastern suburb of Beirut, 300 m altitude) and Chehim (40 Km South of Beirut, 500 m altitude). Nine and five ovitraps were placed in each location respectively. Each trap consists of a black bucket containing 500 mL of water and a rectangular wooden strip where mosquitoes lay their eggs. Traps were placed in fixed locations during the period extending from April 2015 to December 2015 and were monitored weekly for egg collection. The number of eggs was counted under a stereomicroscope and the average number of eggs per trap and per week was calculated for each location.

### Monitoring insecticide resistance: susceptibility bioassay

Mosquito eggs from Fanar location were hatched in order to obtain larval then adult stages. Susceptibility tests were applied on adult specimens following WHO standard bioassay [24]. Adults were exposed to diagnostic doses of the following insecticides as recommended for *Ae*. *albopictus* and, when not available, for *Ae*. *aegypti* [24,25]: DDT 4% (Organochlorines), Malathion 0.8% (Organophosphates), Propoxur 0.1% (Carbamates), Permethrin 0.25% (Pyrethroids type I), Lambda cyahlothrin 0.03% (Pyrethroids type II).

Insecticide-impregnated papers at the indicated doses were provided by a WHO collaborating center (IRD-MIVEGEC, Montpellier-France). For each chemical, two to four batches of around 25 young (3 to 5 days) non blood-fed females were exposed to impregnated papers in exposure tubes for one hour. Afterward, mosquitoes were transferred to recovery tube, fed with 10% glucose solution and maintained at 26°C ± 2°C and 80% humidity. For each insecticide test, two to three control batches of 25 mosquitoes were used; they were exposed to papers impregnated with acetone mixed with silicone oil, solvent used to prepare the insecticide formulation. Mortality for test and control batches was recorded 24 hours post-exposure.

### Vector competence to ZIKV

#### Mosquito collection

Mosquito eggs collected in 2015 from Fanar location were sent to the Laboratory of Arboviruses and Insect Vectors (Institut Pasteur Paris) for vector competence assessment.

Eggs were hatched and obtained larvae were reared at 26 ±1°C in pans of 200 individuals and fed every 2 days with a yeast tablet dissolved in 1L of dechlorinated tap water. Emerging adults were morphologically identified and only *Ae*. *albopictus* mosquitoes were maintained at 28±1°C with a 12L:12D cycle, 80% relative humidity and supplied with a 10% sucrose solution. Females were fed twice a week on anaesthetized mice (OF1 mice, Charles River laboratories, France). Resulting F2 adults were used for vector competence assays.

#### Mosquito experimental infections

ZIKV strain (NC 2014–5132) isolated from a patient in 2014 in New Caledonia was used in the competence experiments. This strain belongs to the same genotype as the one that circulated in Brazil in 2015 [26]. Batches of 60 7–10 day-old females were exposed to an infectious blood meal containing 1.4 mL of washed rabbit erythrocytes and 700 μL of ZIKV suspension at a titer of 10^7.2^ plaque-forming unit (pfu)/mL for ZIKV. ATP was added as a phagostimulant at a final concentration of 1 mM. Mosquitoes were allowed to feed for 15 min through a pork intestine using the Hemotek feeder maintained at 37°C. Fully engorged females were transferred in cardboard containers and maintained with 10% sucrose under controlled conditions (28±1°C, relative humidity of 80%, 12L:12D cycle) for up to 28 days with mosquito analysis at 7, 14, 21 and 28 days post-infection (dpi). For each virus, 21–44 mosquitoes were examined at each dpi.

#### Infection and dissemination assays

In order to determine the infection rate, the body of each female (abdomen and thorax) was tested at 7, 14, 21 and 28 dpi. For that, the body was ground in 250 μL of Leibovitz L15 medium (Invitrogen, CA, USA) supplemented with 3% FBS, and centrifuged at 10,000×g for 5 min at +4°C. The supernatant was processed for viral titration. Similar processing was performed for mosquito heads in order to determine the dissemination efficiency. Infection rate (IR) refers to the proportion of mosquitoes with an infected body (proxy of an infected midgut) among examined mosquitoes and dissemination efficiency (DE) to the proportion of mosquitoes with an infected head (virus able to disseminate from the midgut to the head) among tested mosquitoes.

#### Transmission assays

Previously analyzed females were also tested for viral transmission by collecting their saliva using the forced salivation technique as previously described [27]. Mosquitoes were anesthetized on ice and legs and wings were removed. Saliva was then titrated to estimate the transmission efficiency (TE) which corresponds to the proportion of mosquitoes with infectious saliva (virus excreted from saliva, likely inoculated into a vertebrate host) among tested mosquitoes.

#### Viral titration

ZIKV was titrated by plaque forming assay as described previously [28]. Serially diluted body and head homogenates were inoculated onto monolayers of Vero cells in 96-well plates. Following incubation for 7 days at 37°C, cells were stained with a solution of crystal violet (0.2% in 10% formaldehyde and 20% ethanol). The presence of viral particles was assessed by CPE detection. Similarly, saliva was titrated on monolayers of Vero cells in 6-well plates incubated 7 days under an agarose overlay. Saliva was considered infected when at least one viral particle was detected.

#### Statistical analysis

Means, standard deviations, 95% confidence interval were calculated and statistical analyses were performed using the Stata software (StataCorp LP, Texas, and USA). The effect of virus and dpi on infection, dissemination and transmission rates was evaluated using Fisher’s exact test. The titer of viral particles in mosquito saliva was compared across groups using a Kruskall-Wallis non-parametric test. P-values<0.05 were considered statistically significant.

## Results

### Mapping of *Ae*. *albopictus* in Lebanon

**Fig 1:**
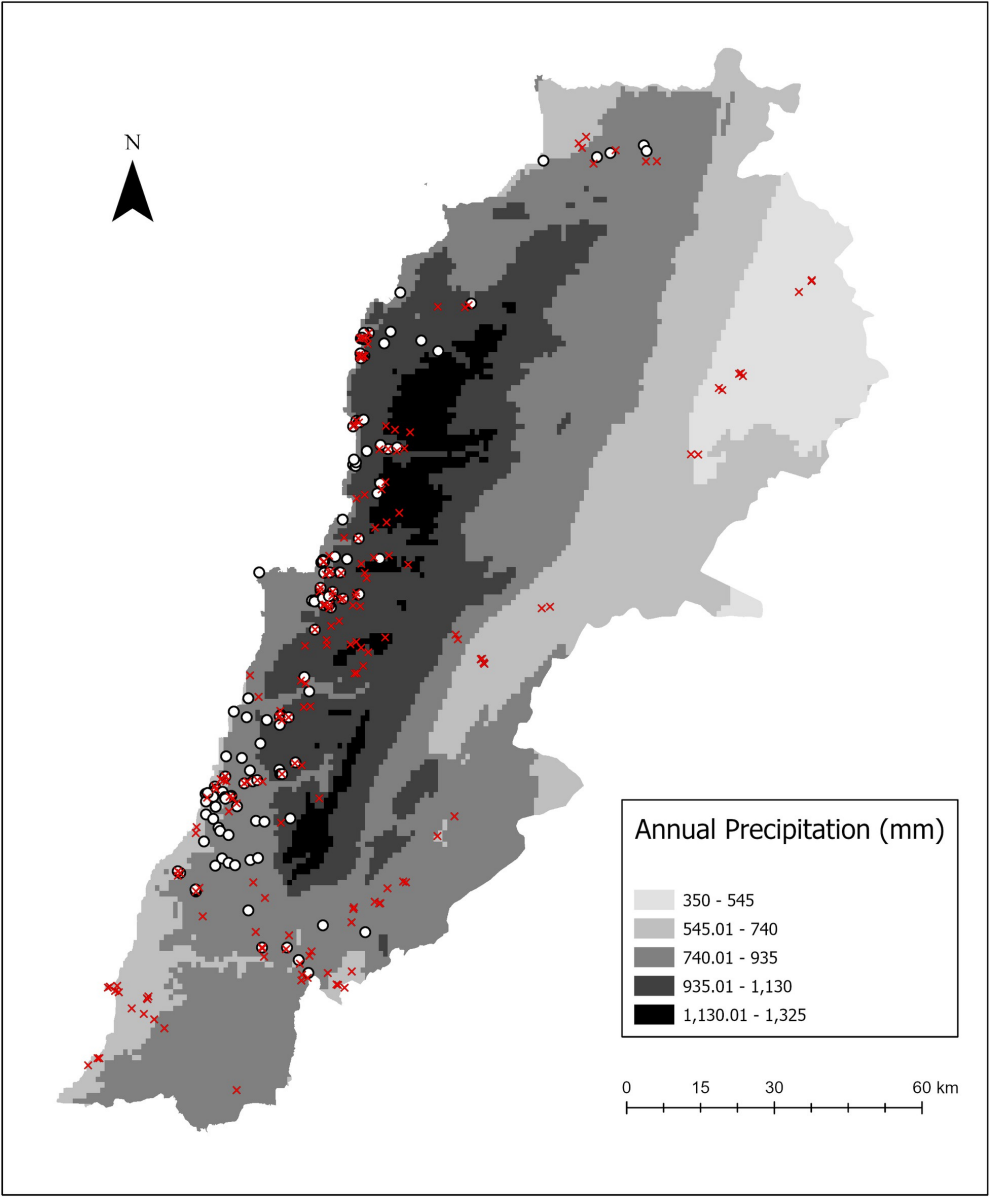
Average annual precipitation map showing collection points of *Ae*. *albopictus* in Lebanon during 2015 and 2018. White dots and red marks represent presence and absence locations respectively. Precipitation layer source: Fick, S.E. and R.J. Hijmans, 2017 (https://doi.org/10.1002/joc.5086). Basemap layer source: Esri. "Human Geography" [basemap]. 1:967380. "Human Geography Base". Nov 3, 2017. https://www.arcgis.com/home/item.html?id=2afe5b807fa74006be6363fd243ffb30. (March 3, 2021).

A total of 416 collection points were screened for the presence/absence of *Ae*. *albopictus* in a wide range of geographic locations covering Lebanese territories. Collections yielded 167 presence (40.14%) and 249 absence points that were plotted on the country map on a layer of average annual precipitation (Fig 1). This map showed large spreading of *Ae*. *albopictus* mainly on the western versant of the Mount Lebanon chain characterized by relatively important humidity and precipitation levels. It was also recorded inland at the northern and southern border regions. However, *Ae*. *albopictus* was absent from arid and semi-arid areas of the Bekaa valley (Zahle, Baalbeck and Hermel districts) where precipitation levels are relatively low (Fig 1). Altitude data showed that *Ae*. *albopictus* was present from the sea level up to 1000 m.

### Seasonal dynamics of *Aedes albopictus*

**Fig 2:**
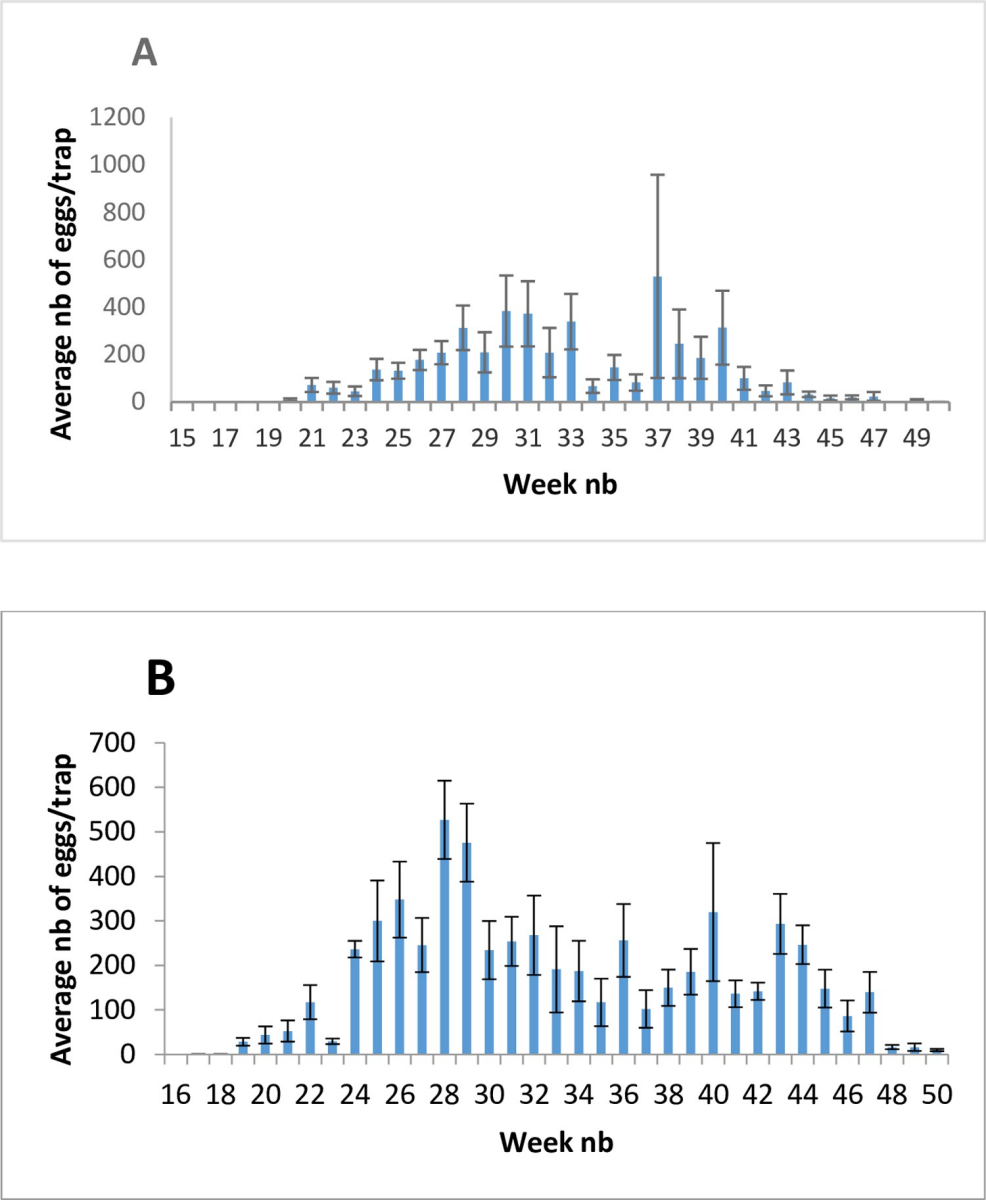
**Seasonal dynamics of oviposition activity of *Ae*. *albopictus* in Lebanon represented by the average number of laid eggs per trap and per week in Chehim (A) and Fanar (B) localities.** Error bars represent standard error of the mean.

A total of 75,014 eggs were collected during 2015. The average numbers of collected eggs per trap and per week in Fanar and Chehim locations showed that oviposition activity started at week 18 and 19 respectively. This activity increased during the summer season and continued during the fall. It remained detectable until December (week 50) in both locations when the monitoring ceased. Two sharp peaks of oviposition were observed in both locations (in addition to two other smaller peaks). The first was in July on weeks 28 and 30 respectively. The second peak was on week 40 and 37 respectively and was markedly higher in Chehim during which an average of 530 eggs per trap and per week was recorded. A noticeable drop in oviposition was observed between both peaks during the second half of August in both locations (Fig 2). However, the low number of monitored traps (9 in Fanar and 5 in Chehim) and the important variation in the number of laid eggs/week for each of these traps (as reflected by the important length of certain standard error bars) make this bimodal distribution dubious.

### Susceptibility bioassays to insecticides

Insecticide bioassays showed that the *Ae*. *albopictus* population tested was highly susceptible to Permethrin and to Lambda-cyahlothrin. The observed mortality rate was 100% and 98.63% respectively. Both chemicals belong to pyrethroids, a widely used group of insecticides. Besides, exposure to Propoxur (carbamates) revealed a mortality rate of 96.46% suggesting a suspected resistance according to the WHO guidelines [24]. *Ae*. *albopictus* was resistant to Malathion (organophosphates) and to DDT (organochlorines); mortality rates were respectively 25.33% and 81% following 1 h exposure to each of these chemicals (Table 1).

**Table 1:** Mortality rate following bioassays using DDT 4% (organochlorines), Malathion 0.8% (organophosphates), Propoxur 0.1% (carbamates) and Permethrin 0.25% and Lambda-cyhalothrin 0.03% (pyrethroids).

		Insecticides				
		Organochlorines	Organophosphates	Carbamates	Pyrethroids Type I	Pyrethroids Type II
		DDT 4%	Malathion 0.8%	Propoxur 0.1%	Permethrin 0.25%	Lambda-cyahlothrin 0.03%
**Exposed specimens**	No of tested specimens	53	75	85	96	73
	No of killed specimens	43	19	82	96	72
	Observed Mortality (%)	**81.13**	**25.33**	**96.47**	**100**	**98.63**
**Control specimens**	No of control specimens	53	49	76	42	54
	No of killed	0	1	2	0	2
	Observed Mortality (%)	0	2.04	2.63	0	3.7

### Vector competence

**Fig 3:**
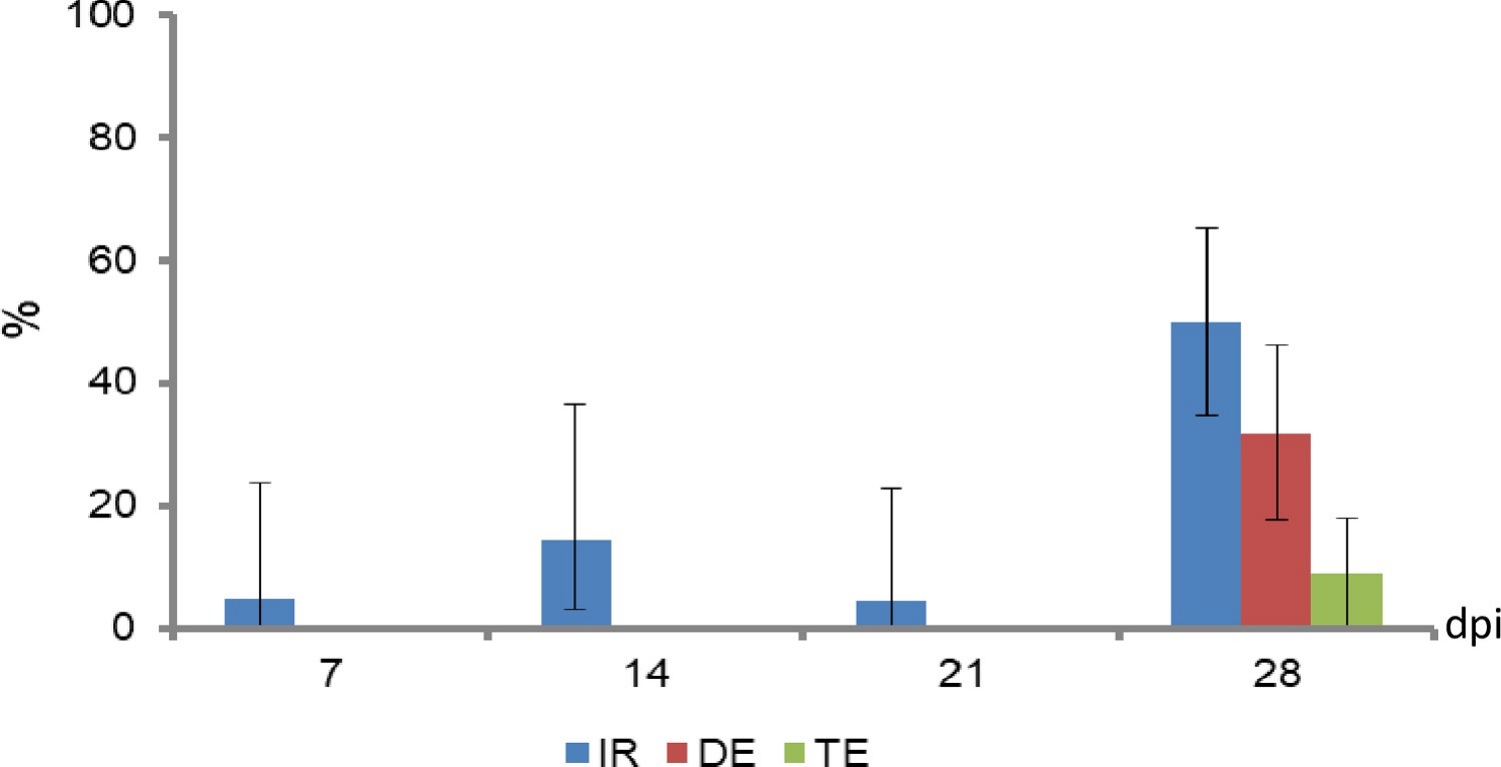
Vector competence of *Ae*. *albopictus* following exposure to a ZIKV-infected blood meal provided at 10^7.2^ pfu/mL. Infection rate (IR) refers to the proportion of mosquitoes with infected midgut among tested ones (alive and engorged at Day 0). Dissemination efficiency (DE) corresponds to the proportion of mosquito females with infected head/legs or wings among tested ones. Transmission efficiency (TE) relates to the proportion of mosquito females with infectious saliva among tested ones.

Following exposure to ZIKV-infected blood meal, only 4.75% (N = 21) of engorged females were infected at 3 dpi. This rate remained relatively low at 14 and 21 dpi to reach 50% (N = 44) at 28 dpi (Fig 3). Viral dissemination and transmission occurred only at 28 dpi. Four of the tested females (9%, N = 44) were able to excrete viral particles in their saliva, a crucial condition for viral transmission (for details, see S1 Table).

## Discussion

The introduced populations of the tiger mosquito obviously succeeded to adapt to local environmental conditions and colonized the coastal band and western versant of Mount Lebanon. *Aedes albopictus* was first reported in Lebanon in 2003 [15], when only 11 larvae had been identified in 4 of 150 inspected breeding sites. Three sites were located on the Western versant of Mount Lebanon chain and one in Hermel, in an arid zone North the Bekaa valley. Two decades after these first records, we observe an important geographic expansion of this invasive mosquito in the humid and sub-humid zones along the coastal band and on the heights of the Mount Lebanon where major urban agglomerations are located. These agglomerations offer obviously more larval habitats compared to rural areas as was observed in several countries including China where larvae development and adult emergence rates were clearly higher in urbanized areas than in rural areas [29,30]. Nevertheless, the tiger mosquito apparently failed to establish in the arid and semi-arid zones in Lebanon. Despite its introduction to Hermel region in 2003, no specimen of *Ae*. *albopictus* were collected during 2015 and 2018 from this region and from the whole arid zone of the Bekaa valley. Several environmental factors can be determinant. Absolute humidity can restrict mosquito settlement in dry areas but favor it in coastal zones where vector introduction is highly likely due to greater human flow and trade activities [31]. Likewise, temperature (annual mean temperature and the coldest month mean temperature) is considered critical for the settlement of the tiger mosquito [32,33]. In many countries, this mosquito showed important ecological plasticity and was capable to adapt to different types of breeding sites when shifting from wet to dry seasons [34]. Besides, it was observed in different ecosystems, and like in Lebanon, at different altitudes reaching 1200 m in La Reunion Island [35] and in Albania [33].

Oviposition monitoring showed that *Ae*. *albopictus* is active during most of the year (Fig 2) which represents a risk for local transmission of related arboviruses, such as Zika virus but also for Chikungunya and Dengue viruses[23]. Indeed, these viruses can be potentially introduced to Lebanon through the several thousands of Lebanese expatriates in African and South American countries that return home to spend their vacations. The bimodal oviposition pattern is observed in several Mediterranean countries such as Croatia and Italy. Nevertheless, peak weeks differs between countries and may change between years. In Lebanon, oviposition started at the beginning of the spring and lasted 30 to 32 weeks. It has been suggested that the initiation of seasonal oviposition activity is triggered by the rise of mean temperature while its decline is stimulated by the decrease of daylength below a critical value which will result in laying diapausing eggs [30,36,37]. Tracking female oviposition activity is useful to decide control strategies as was validated by several authors [30,38].

Exposure of Lebanese strains of *Ae*. *albopictus* to insecticides of the four major groups showed that these mosquitoes were sensitive to pyrethroids and to carbamates however clearly resistant to DDT (organochlorines) and to Malathion (organophosphates). The use of organochlorines is officially banned in Lebanon since 1982 [39]. Resistance to DDT detected in this study can be explained by the illegal use of this insecticide. Several monitoring studies reviewed by Helou and collaborators (2019) [39] revealed the presence of DDT and its metabolites in environmental (water and soil) and human (breast milk and sera) samples. Moreover, it is also likely that the observed resistance is due to an inherited gene carried by the first introduced specimens to Lebanon two decades ago most likely from Europe or North America as suggested by phylogenetic studies [23]. Resistance to DDT has been documented in several countries mainly in Asia but also in Europe and America [13]. In some regions, this resistance was partially attributed to cross resistance with organophosphates [40] which warrants further investigations.

Resistance to malathion was markedly high. This insecticide in addition to other organophosphates including chlorpyrifos and dichlorvos, are heavily used for mosquito control in Lebanon despite the significant shift towards the use of pyrethroids in 2006 [41]. Resistance to organophosphates was also detected in Lebanese populations of *Culex pipiens* collected in 2005 that had resistant *ace-1* alleles carrying the G119S and F290V substitutions [41]. Resistance to the organophosphates, less common than to DDT, has also been recorded in *Ae*. *albopictus* populations in Southeast Asia, Pakistan and America [13,42]. Recently *Ae*. *albopictus* populations from several provinces in Greece, displayed high resistance to malathion [43].

Tested populations of *Aedes albopictus* did not show high vector competence to ZIKV according to the indices IR, DE and TE. Despite high virus titer used for infection (7.2 log10 FFU/mL), ZIKV was shed into saliva only by 28 dpi. Such long extrinsic incubation period, equivalent to the female lifespan, negatively impacts the vector capacity of the mosquito and decreases significantly the transmission risk. In this study, ZIKV infects the midgut starting at 7 dpi. However, the apparent inability of the virus to disseminate at 7, 14 and 21 dpi suggests the possibility of a midgut escape barrier. Using other ZIKV variants (from Senegal, Mexico and Brazil), it has been shown that the salivary glands barrier limited or even completely prevented ZIKV from being shed into saliva [44] whereas the intestinal barrier appeared relatively more permissive. For those variants, when provided in blood meals at comparable viral titers, dissemination was observed as early as 3 dpi [44]. This could be related to mosquitoes’ microbiome [45] or even to virus-vector molecular interactions [45].

Vector competence of the tiger mosquito to ZIKV varies with the geographic origin. In a meta-analysis involving 23 studies on *Ae*. *albopictus* from different geographic origins, McKenzie and collaborators [6] observed that European, North and South American strains of this mosquito had high infection rates for ZIKV comparable to those of *Ae*. *aegypti*, the primary vector of ZIKV [46]. However, transmission rates for ZIKV were obviously low in certain regions mainly in Europe [6]. Consequently, the risk of ZIKV transmission in Europe is considered minimal [47,48]. Despite this reduced risk, autochthonous vector-borne transmission of ZIKV, involving only three cases, occurred in the Var department, south of France [12]. Obviously other factors such as vector longevity, feeding behaviors and vector-host exposure may compensate low vector competence and allow transmission to occur.

Lebanese populations of *Ae*. *albopictus* are less suitable for ZIKV transmission compared to CHIKV and DENV. In fact, 30% of tested mosquitoes were able to deliver CHIKV particles in the saliva at 10 dpi and 38% of them delivered DENV-2 at 21 dpi [23]. Nevertheless, congruent environmental factors may trigger local transmission especially when considering the important numbers of expatriates returning to Lebanon from endemic South American countries.

This study underlines important geographic spread of the tiger mosquito in Lebanon especially to humid regions on the western versant of the Mount Lebanon. This mosquito is active for at least 8 months per year with two peaks during the summer season. Moreover, Lebanese populations of *Ae*. *albopictus* are susceptible to pyrethroids and carbamates but resistant to organochlorines and organophosphates. Nevertheless, further susceptibility testing is needed using revised doses of adulticides established specifically for *Ae*. *albopictus* to avoid a misestimation of insecticide susceptibility which are key in setting up appropriate control programs against this mosquito. Such programs should be considered a health priority to prevent potential autochthonous transmission of arboviruses for which environmental factors and mosquito vector competence are obviously favorable.

## Supporting information

S1 Table Details on infection, dissemination and transmission of *Aedes albopictus* 7, 14, 21 and 28 days after an infectious blood meal provided at a titer of 10^7.2^ pfu/mL. (DOCX)
