# Supplementary material for: The tiger mosquito in Lebanon two decades after its introduction: A growing health concern
Source: PLoS Negl Trop Dis. 2022 Feb 9;16(2):e0010206. doi: 10.1371/journal.pntd.0010206 (PMC8863254; doi:10.1371/journal.pntd.0010206)
Supplement: S1 Table — (DOCX) [file pntd.0010206.s001.docx]

**S1 Table.** Details on infection, dissemination and transmission of *Aedes albopictus* 7, 14, 21 and 28 days after an infectious blood meal provided at a titer of 10^7.2^ pfu/mL.

| Day post-infection | IR | | |  | DE | | |  | TE | | |
| --- | --- | --- | --- | --- | --- | --- | --- | --- | --- | --- | --- |
|  | N tested | N positive | N negative |  | N tested | N positive | N negative |  | N tested | N positive | N negative |
| 7 | 21 | 1 | 20 |  | 21 | 0 | 21 |  | 21 | 0 | 21 |
| 14 | 21 | 3 | 18 |  | 21 | 0 | 21 |  | 21 | 0 | 21 |
| 21 | 22 | 1 | 21 |  | 22 | 0 | 22 |  | 22 | 0 | 22 |
| 28 | 44 | 22 | 22 |  | 44 | 14 | 30 |  | 44 | 4 | 10 |

IR (infection rate) refers to the proportion of mosquitoes with an infected body (proxy of an infected midgut) among examined mosquitoes. DR (dissemination efficiency) corresponds to the proportion of mosquitoes with an infected head (virus able to disseminate from the midgut to the head) among tested mosquitoes. TE (transmission efficiency) corresponds to the proportion of mosquitoes with infectious saliva (virus excreted from saliva) among tested mosquitoes. N tested, total number of tested mosquitoes. N positive, number of infected mosquitoes. N negative, number of non-infected mosquitoes.
